# Agrivoltaic systems have the potential to meet energy demands of electric vehicles in rural Oregon, US

**DOI:** 10.1038/s41598-022-08673-4

**Published:** 2022-03-17

**Authors:** Casey L. Steadman, Chad W. Higgins

**Affiliations:** 1 Department of Biological and Ecological Engineering, Oregon State University, 116 Gilmore Hall 124 SW 26th St, Corvallis, OR USA; 2 Department of Water Resources Science, Oregon State University, 116 Gilmore Hall 124 SW 26th St, Corvallis, OR USA

**Keywords:** Climate sciences, Environmental sciences

## Abstract

Electrification of the transportation industry is necessary; however, range anxiety has proven to be a major hindrance to individuals adopting electric vehicles (EVs). Agrivoltaic systems (AVS) can facilitate the transition to EVs by powering EV charging stations along major rural roadways, increasing their density and mitigating range anxiety. Here we conduct case study analyses of future EV power needs for Oregon, USA, and identify 174 kha of AVS viable agricultural land outside urban boundaries that is south facing and does not have prohibitive attributes (designated wetland, forested land, or otherwise protected lands). 86% highway access points have sufficient available land to supply EV charging stations with AVS. These AVS installations would occupy less than 3% (5 kha) of the identified available land area. Installing EV charging stations at these 86% highway access points would yield 231 EV charging stations with a median range of 5.9 km (3.6 mi), a distance comparable to driver expectations, suggesting that this approach would serve to mitigate range anxiety. AVS powered rural charging stations in Oregon could support the equivalent of 673,915 electric vehicles yr^−1^, reducing carbon emissions due to vehicle use in OR by 3.1 mil MTCO_2_ yr^−1^, or 21%.

## Introduction

The transportation sector contributes roughly 25% of global CO_2_ emissions from fossil fuel combustion^1^ and is the fastest growing source of all greenhouse gas emissions^2^. Passenger vehicles make up the majority (44%) of the transportation sector’s energy demand and approximately 80% of those vehicles are powered by gasoline^3^. Dependence on non-renewable fossil fuels and significant environmental impacts make the current transportation sector unsustainable^2^.

Electric vehicles (EVs) are considered the most promising advancement in the pursuit of sustainable transportation^4^. Widespread adoption has the potential to reduce oil dependency and emissions^5^, particularly when the EV manufacturing process and electricity generation are decarbonized^2,6,7^. This potential has been recognized by governments across the globe, which have set goals that include eliminating gasoline and diesel vehicles and fully converting to electric or hybrid vehicles by as early as 2025^8,9^. While the widespread promotion of EVs has led to significant growth in EV ownership in many countries over the past decade^10^, the market share of EVs remains small, representing only 4.6% of vehicle sales worldwide in 2020^11^. Further, the projected growth of EV sales varies significantly across regions and countries. The US is projected to reach only 8% of new car sales by 2030, compared to 26% and 28%, respectively, in Europe and China^8^.

A primary hindrance to widespread adoption of EVs is range anxiety, particularly in rural America^12^. Range anxiety is the fear of running out of electricity before reaching a charging station and is the most influential factor when a consumer considers an EV purchase^13^. Two distinct challenges must be addressed to mitigate range anxiety: (1) improve the range EVs can travel on a given charge, and (2) increase the density of charging stations, particularly in rural areas where their density is lowest^12,14^.

The emerging technology of agrivoltaics presents a unique opportunity to improve charging infrastructure in rural areas where electrical infrastructure tends to be weaker^12^. Agrivoltaic systems (AVS) co-locate agricultural production and photo voltaic energy production for mutual benefits. These benefits can include, for some areas and climates, increased agricultural production^15–18^, increased renewable energy production^19,20^, increased land use efficiency^21–24^, and increased total revenue^18,19,25,26^. Further, agricultural land is recognized as the land cover type with the most solar power production potential^20^. Agricultural land is also distributed where EV charging station density is lowest. AVS has the potential to generate clean energy on-site at rurally-distributed points of EV demand without competing for land with the agriculture industry. Here, we evaluate the feasibility of leveraging AVS technology to improve EVCS infrastructure. Through a site suitability analysis, we analyze the rural locations at which AVS is capable of powering EVCSs to meet future EV power needs in magnitude. Temporal matching of load and generation were not considered. Also note that this analysis takes a highly conservative approach. That is the envisioned scenario is maximum demand (traffic) on calendar month with lowest photovoltaic generation potential. Our results show that even within these constraints, agrivoltaics could play a role in charging station infrastructure development. We evaluate the potential for our proposed approach to mitigate range anxiety and CO_2_ emissions related to vehicle use.

## Results

**Figure 1:**
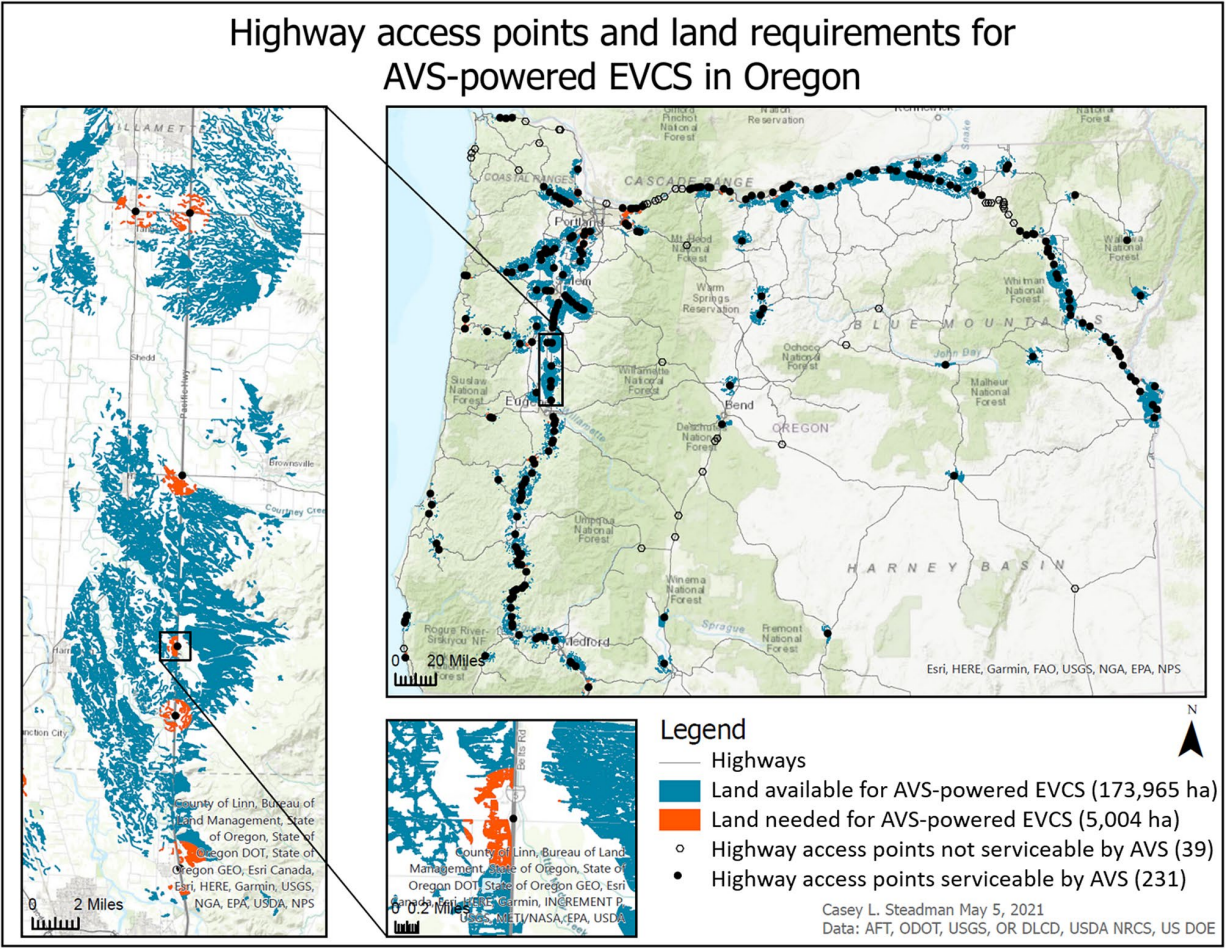
Model results with distribution and quantity of highway access points serviceable by AVS, highway access points not serviceable by AVS, land supply available, and portion of land supply needed to meet electricity demand at serviceable highway access points.

The distribution and quantity of highway access points and land is shown in Fig. 1. Blue land represents total land area that satisfies suitability criteria for AVS installation: farmland with soil classes 3 or 4 and southern-facing aspects located within 5 miles of rural highway access points. The total land supply is 174 kha. A total of 270 rural highway access points was identified. Of those highway access points, 231 (86%; Table 1) had sufficient land area to service EVCSs with AVS. These areas are indicated with black circles (Fig. 1). To meet the very conservative estimate of EVCS electricity demand (ensuring that enough energy is available for every car to reach the next charging station irrespective of prior charging decisions) at the identified 86% of highway access points throughout OR, 5 kha (2.9%) would be required. These areas are indicated in orange (Fig. 1). 39 highway access points could not utilize AVS, indicated by hollow hexagons.

**Table 1:** Summary of model results reflecting total number of highway access points in the network, portion and percent of those highway access points serviceable by AVS; total land supply available, portion and percent of land supply needed to power serviceable highway access points in the network; total annual CO_2_ emissions in Oregon from passenger vehicle use and the portion and percent of those annual emissions this approach has the potential to reduce; and total passenger vehicles registered in Oregon and the equivalent number of vehicles and percent this approach has the potential to support as electric vehicles.

Variable	Total	Portion	Percent (%)
Highway access points (count)	270	231	86
Land (ha)	173,965	5004	3
CO_2_ (mil MTCO_2_ yr^−1^)	15	3.1	21
Vehicles (count)	3,200,000	673,915	21

Of the 231 AVS-serviceable highway access points, 220 (95%) have a distance between them that is less than 27 km (17 mi). There is considerable variability in range and distances lie between 0.56 km (0.75 mi) and 45 km (28 mi) with one outlier of 135 km (84 mi). Including the outlier, the mean distance is 8.9 km (5.5 mi) with a standard deviation of 11.4 km (7.1 mi). While recognizing that preferences may vary as the autonomy of EVs continues to improve, the range distribution values can be put in the context of potential EV consumer preferences. Pevec et al.^27^ conducted a survey of such preferences based on the area in which the potential EV consumer lives, from large metropolitan areas to villages. They found that the mean preferred distance between available charging stations was 4.1 km (2.5 mi) for those living in a metropolis and 10.6 km (6.6 mi) for those living in a town. The overall preferred distance between charging stations to reduce range anxiety was less than 5 km (3.1 mi). Our central tendency values fall within this range of preferred distances. However, this survey was conducted in Croatia and a similar survey has not been conducted in the US to our knowledge.

A network range comparison can also be made to current gas station infrastructure. An analysis of the distances a driver must divert from their route to refuel in large metropolitan areas in the US was summarized Melania et al.^28^. Mean diversion distances were organized by how closely clustered the gas stations were and ranged from approximately 4 km (2.5 mi) to approximately 29 km (18 mi) with distance decreasing as clustering increased. Again, our central tendency values fall within this range.

In OR, 3.2 mil passenger vehicles are registered^29^ and the great majority of those vehicles are internal combustion engine vehicles^30^ which collectively emit approximately 15 mil MTCO_2_ each year^31^. The total CO_2_ reduction potential of AVS-serviced EVCSs was estimated to be 3.1 mil MTCO_2_ (21%) if our approach were fully implemented; equivalent to 673,915 vehicles each year.

## Discussion and conclusions

Here we have shown that servicing rural EVCSs in OR with AVS is feasible, requiring only 3% of total land supply to power 86% of rural highway access points throughout the state (Table 1). This is significant because rural areas may not have the requisite grid transmission infrastructure to support EV charging stations, and agrivoltaics would shift energy production to the point of use. Drivers could anticipate access to charging stations at the great majority of highway access points in rural OR if this approach were implemented. Oregon currently has 670 EVCSs^30^. The addition of 231 EVCSs would supplement that value by 34%. Further, distance between EVCSs may fall within preferred ranges of travel of the EV consumer. Therefore, the network density may be sufficient to alleviate range anxiety. Finally, this approach has the potential to reduce annual carbon emissions from passenger vehicle use in OR by 21% by providing clean energy to the equivalent of 673,915 vehicles each year.

## Methods

**Figure 2:**
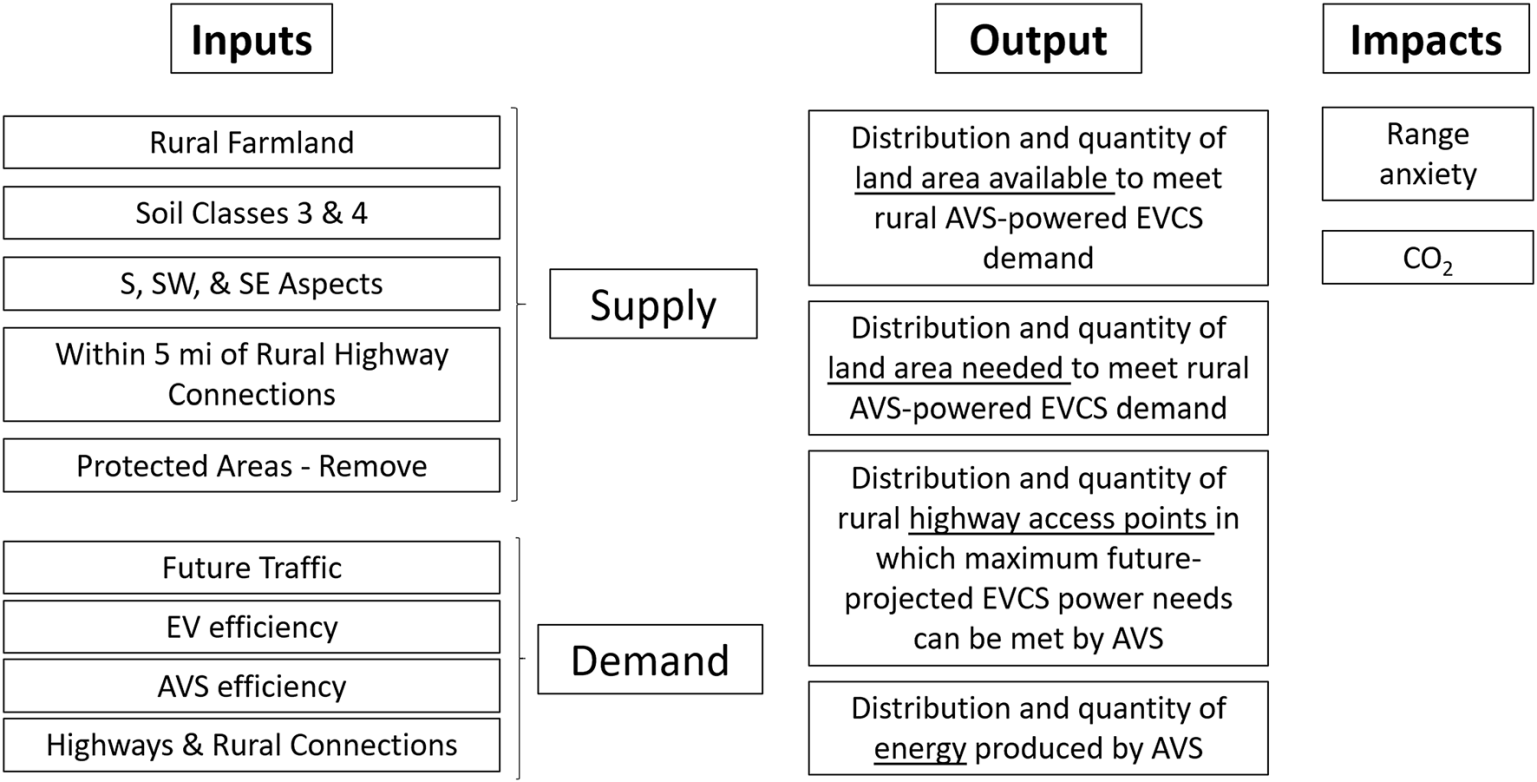
Analytical model inputs, outputs, and impacts.

Our model evaluates the distribution and quantity of: (1) land available (supply) to meet rural AVS-powered EVCS demand; (2) land area needed (demand) to meet projected rural AVS-powered EVCS demand; (3) rural highway access points where EVCS power needs can be met by AVS; (4) total power production potential. Model parameters are summarized in Fig. 2. Data inputs are summarized in Table 2. We applied the model to the state of Oregon, USA to evaluate the potential of AVS-powered EVCSs at rural highway access points throughout the state to create charging access when a driver enters or exits a major highway.

**Table 2:** Data inputs for analytical model.

Input data	Source
Highways, traffic	Oregon Department of Transportation, 2019
Urban growth boundaries	Department of Land Conservation and Development, 2019
Farmland	American Farmland Trust, 2016
Soils	US Department of Agriculture, Natural Resources Conservation Service, 2019
DEMs	US Geological Survey, 2019
Floodplains	The Wetlands Conservancy, 2009
Lakes, ponds, rivers	US Geological Survey, National Geospatial Technical Operations Center, 2020
Riparian habitat	Oregon Department of Fish and Wildlife, 2016
State parks	Oregon Parks and Recreation Department, 2018
Protected areas	Institute for Natural Resources, 2015
Top selling electric vehicles	US Department of Energy, 2020
Solar radiation	US Geological Survey, National Renewable Energy Lab, 2019
Agrivoltaic system efficiency	Dinesh and Pearce, 2016

We projected all data layers to NAD 1983 Oregon Statewide Lambert (Intl Feet). We conducted analysis in ESRI ArcGIS Pro 2.7.0 and Python. We assumed urban areas would have power supply options available for EVCS and would have EVCSs available. Therefore, we excluded land and highway connections within urban growth boundaries (UGBs) from analysis. We assumed that travel within the area of UGBs to access an EVCS would be adequately represented by estimating travel to the centroid of each UGB polygon which we snapped to the major highway.

### Supply

We defined the land supply as the land area (m^2^) available for agrivoltaic systems (AVS) meeting a set of criteria including location, land characteristics, and compliance with government regulations. The total supply of available land area was restricted to lie within an 8 km (5 mi) radius of rural highway access points in accordance with the West Coast Electric Highway^32^ documentation that recommends the same. Rural highway access points were identified as connections along major highways^33^ located outside of urban growth boundaries^34^ (UGB) that were located a minimum of 0.8 km (0.5 mi) from the next nearest rural highway connection. Highway connections with proximity less than 0.8 km (0.5 mi) were merged and represented as a single access point within the analysis. All highway connections located within UGBs were excluded.

Land AVS potential was further restricted based on the following attributes. The land must be designated as farmland indicated by the American Farmland Trust (AFT) productivity, versatility, and resiliency (PVR) scale^35^. Only lands with soil classes 3 and 4^36^ were considered in compliance with Oregon land use regulations prohibiting solar development on class 1 and 2 soils^37^. Only land with S, SW, and SE aspects as calculated from 10 m digital elevation models^38^ (DEM) to maximize AVS productivity were included. We also ensured no land was included as supply that was a designated floodplain^39^, lake, pond, or river^40^, riparian habitat^41^, state park^42^, natural protected area^43^, or other protected areas including tribal lands^44^. Finally, we removed any land parcels with areas less than 1 ha.

**Figure 3:**
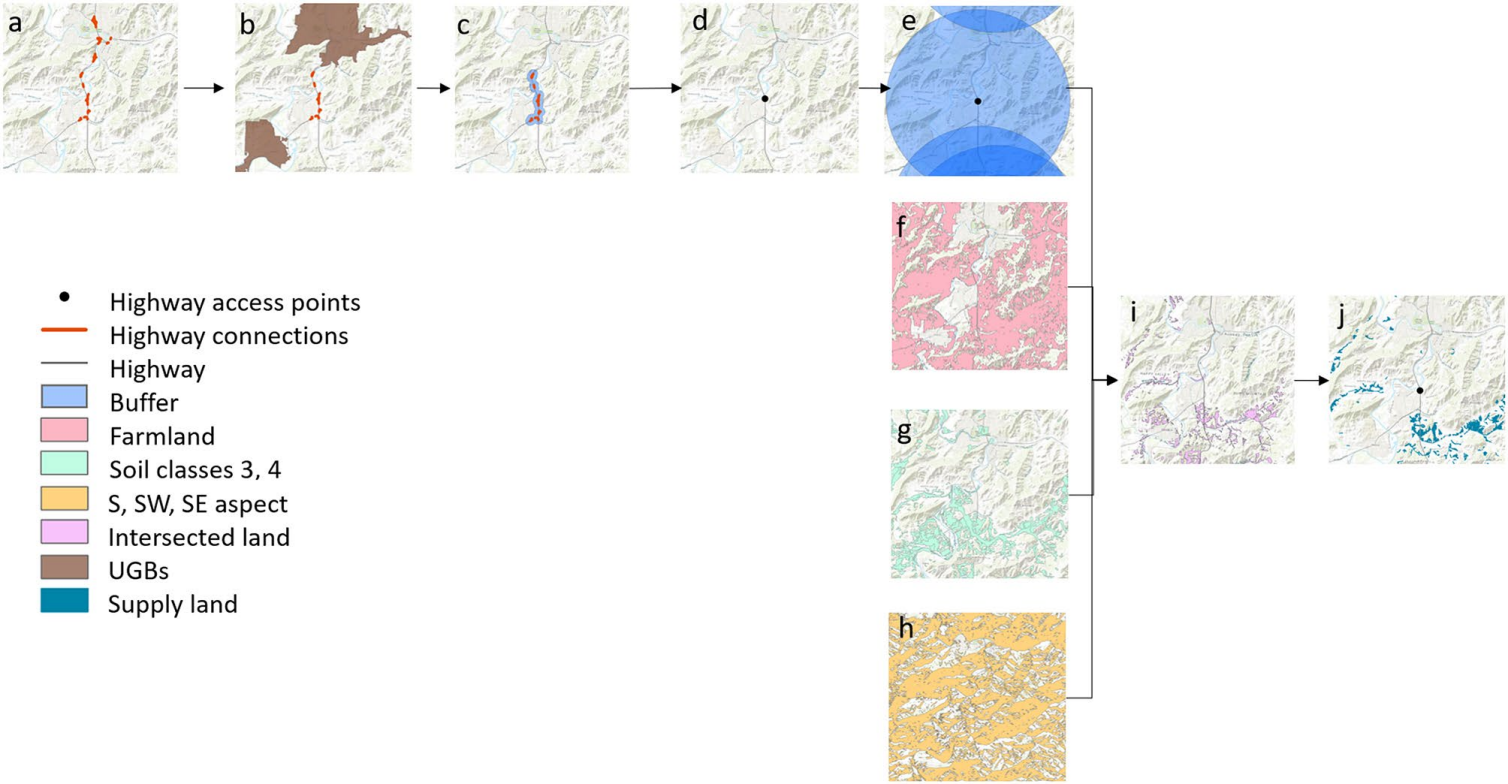
Analytical steps in creating land supply layer. (**a**) highway network with highway connections in orange, (**b**) urban growth boundary (UGB) polygons in brown and highway connections within UBGs erased, (**c**) dissolved 0.8 km (0.5 mi) buffer around remaining highway connections, (**d**) centroid of highway connections polygons snapped to highway to create highway access points, (**e**) 8 km (5 mi) buffer around highway access points, (**f**) farmland, (**g**) soil classes 3 and 4, (**h**) S, SW, and SE aspects, (**i**) intersection of land in (**e**–**h**), (**j**) final supply layer with land not suitable for AVS removed.

The method is exemplified in Fig. 3. Rural highway access points were identified as follows: We began with the highway data layer (Fig. 3a), and identified all locations labeled as highway connections. Next, we erased all connections within urban growth boundaries, depicted in Fig. 3b. This resulted in subsets of locations that may be proximal (closer than 0.8 km (0.5 mi)). These proximal connections were aggregated (Fig. 3c) and represented as single charge station locations (Fig. 3d). Access points closer than 0.8 km (0.5 mi) were merged. Then, we identified all land area within a linear distance of 8 km (5 mi) to an access point (blue area in Fig. 3e).

**Figure 4:**
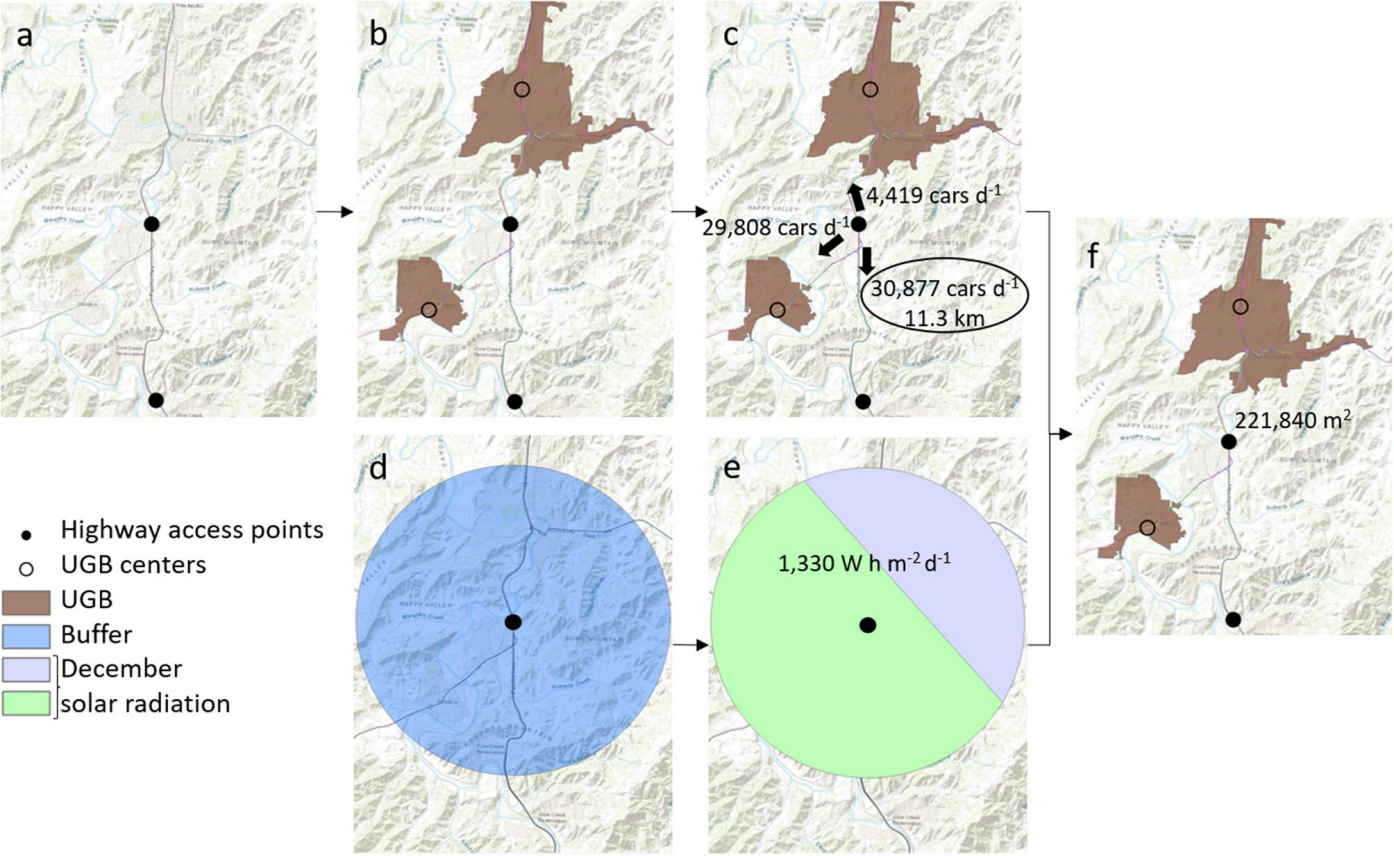
Analytical steps in creating demand layer. (**a**) highway access points, (**b**) centroid of urban growth boundaries (UGB) snapped to highway and color-coded, 20-year average annual daily traffic (AADT), (**c**) weighted average AADT in all possible routes from a highway access point with the maximum value and corresponding distance selected, (**d**) 8.8 km (5.5 mi) buffer around highway access points, (**e**) clipped solaration raster and corresponding weighted average of December solar radiation, (**f**) final demand in areal units.

The total land area within 8 km (5 mi) of each access point was further restricted to generate the supply layer by intersecting this layer with three additional polygon data layers: (1) farmland (Fig. 3f), (2) soil classification (Fig. 3g), and (3) aspect (Fig. 3h). This intersection resulted in Fig. 4i. In the final step we removed any restricted lands, yielding the final land supply layer (Fig. 3j).

### Demand

All calculations of PV energy production and the associated AVS land area calculations are based on the most conservative set of assumptions and criteria: supply all power for EVCSs at rural highway access points during the winter solstice (the time of year when the least solar radiation is available). Thus, the estimates outlined herein represent an upper bound on the total AVS area needed and could be diminished with partial grid connectivity and/or on-site batteries. We quantified demand at each rural highway access point using routes with the maximum weighted average of 20-year projected average annual daily traffic^33^ (AADT), average EV efficiency of the top five selling EVs in the US in 2019^45^, and average AVS efficiency^19,46^. Calculations are described in detail below.

The energy demand is estimated with the following approach visualized by the example in (Fig. 4). The starting location is set at each rural highway access point as shown in the data layer (Fig. 4a). We assume that sufficient energy must be supplied to ensure that all traffic on each route from the EVS charging location can reach the next charging station. That is, the road network may directly connect more than two adjacent EVCS, and when the road network supplies multiple choices for the next charging station, we select the route with the largest energy demand, considering traffic volume and distance. For example, three potential routes to the next charging availability are shown in Fig. 4b. Two of these routes, the routes to the nearest towns (brown polygons) are discarded in favor of the route with greater traffic volume to the South (Fig. 4c). We calculated demand as total AVS land area required to power future EV electricity needs for each point in the rural highway access point data layer using Eq. (1)

1 $$D= {\varepsilon }_{EV} {\varepsilon }_{AVS} \overline{{R }_{s}} Td$$

where *ε*_*EV*_ is mean EV efficiency^45^ (W day car^−1^ mile^−1^), *ε*_*AVS*_ is AVS efficiency^19^ (m^2^ W^−1^), $\overline{{R }_{s}}$ is the local mean daily incident solar radiation on a horizontal surface^46^ (W hr m^−2^ day^−1^), *T* is maximum weighted average of 20-year AADT^33^ (cars day^−1^), *d* is route total distance (miles), and *D* is area of AVS land needed to meet EV power demands (m^2^).

The EV efficiency is the mean efficiency of the top five selling EVs in the US in 2019^45^, *ε*_*EV*_. We estimated the incident solar radiation^46^, $\overline{{R }_{s}}$, to reflect the spatial variability of solar radiation in Oregon, and within the 8.9 km (5.5 mi) radius buffer zone (Fig. 4d) that surrounds each rural highway access point. Solar radiation data points were area weighted averaged according to the area within this buffer zone (Fig. 4e). The conservative AVS efficiency, ${\varepsilon }_{AVS}$, was estimated from literature values to be 13.5%, for a fixed tilt configuration^19^. We then calculated total demand, *D*, by applying these inputs to Eq. (1) (Fig. 4f).

To determine if supply was sufficient to meet demand at every rural highway access point, we iterated through progressively larger buffer sizes in increments of 3 m (10 ft) to a maximum of 8 km (5 mi) for each individual rural highway access point. Note that this methodology does not incorporate property lines. Within each iteration, we then compared the total supply area to the total demand area, *D*. We progressed to the next larger buffer size if supply area within the current iteration was less than the demand area, *D*. The iteration was halted when the circumscribed supply area was equal to or greater than the demand area. If, upon reaching a buffer radius of 8 km (5 mi), the circumcised supply area remains smaller than the demand area, the access point is classified as invalid for AVS.

CO_2_ Emissions Reduction Potential: We calculated CO_2_ emissions reduction potential using Eq. (2)

2 $${R}_{CO2}= \sum {E}_{pt} G C$$

where *E*_*pt*_ is electricity produced at each point (Wh yr^−1^), *G* is the energy conversion factor for gasoline engines (gal Wh^−1^; 0.405 L-gasoline kWh^−1^)^47^, *C* is a conversion to CO_2_ (MTCO_2_ gal^−1^; 8887 g-CO_2_ gal^−1^)^31^, *and R*_*CO2*_ is total CO_2_ reduction potential (MTCO_2_ yr^−1^). We then calculated the total equivalent number of vehicles that could be powered by AVS using Eq. (3)

3 $${{\mathrm{V}}_{\mathrm{e}}= R}_{{\text{CO}}_{2}} V$$

where *V* is a conversion to vehicles (vehicle MTCO_2_^−1^; 4.6 MTCO_2_ vehicle^−1^ yr^−1^)^31^ and *V*_*e*_ is vehicle equivalent.

## Data Availability

Data is available upon request.
